# Physical and Mechanical Properties of Polypropylene Fibre-Reinforced Cement–Glass Composite

**DOI:** 10.3390/ma14030637

**Published:** 2021-01-30

**Authors:** Marcin Małek, Waldemar Łasica, Marta Kadela, Janusz Kluczyński, Daniel Dudek

**Affiliations:** 1 Faculty of Civil Engineering and Geodesy, Military University of Technology in Warsaw, ul. Gen. Sylwestra Kaliskiego 2, 01-476 Warsaw, Poland; marcin.malek@wat.edu.pl (M.M.); waldemar.lasica@wat.edu.pl (W.Ł.); 2 Building Research Institute (ITB), ul. Filtrowa 1, 00-611 Warsaw, Poland; d.dudek@itb.pl; 3 Faculty of Mechanical Engineering, Military University of Technology, ul. Gen. Sylwestra Kaliskiego 2, 00-908 Warsaw, Poland; janusz.kluczynski@wat.edu.pl

**Keywords:** by-product waste, packaging waste, glass cullet, macro-polymeric fibre, recycling, eco-efficient concrete, slump cone, compressive strength, flexural strength, splitting strength

## Abstract

In accordance with the principles of sustainable development, environmentally friendly, low-emission, and energy-intensive materials and technologies, as well as waste management, should be used. Concrete production is responsible for significant energy consumption and CO_2_ production; therefore, it is necessary to look for new solutions in which components are replaced by other materials, preferably recycled. A positive way is to use glass waste. In order to determine the effect of a significant glass cullet content on the properties of concrete, glass powder was used as a filler and 100% glass aggregate. The cement–glass composite has low tensile strength and brittle failure. In order to improve tensile strength, the effects of adding polypropylene fibres on the mechanical properties of the composite were investigated. With the addition of 300, 600, 900, 1200, and 1500 g/m^3^ of fibres, which corresponds to 0.0625%, 0.1250%, 0.1875%, 0.2500%, and 0.3125% of cement mass, respectively, flexural strength increased compared with the base sample by 4.1%, 8.2%, 14.3%, 20.4%, and 26.5%, respectively, while the increase in splitting strength was 35%, 45%, 115%, 135%, and 185%, respectively. Moreover, with the addition of fibres, a decrease in slump by 25.9%, 39.7%, 48.3%, 56.9%, and 65.5%, respectively, compared with the reference specimen was determined.

## Highlights

- Recycled macro-polymer fibres were used to improve tensile strength of the cement–glass composite;
- Reduction of the workability of the cement–glass composite with the addition of polypropylene fibres was obtained;
- Slight effect of waste fibres on the compressive strength of the cement–glass composite was determined;
- With the addition of polypropylene fiber, the flexural strength of the composite increased;
- Significant increase in splitting strength for the fibre-reinforced cement–glass composite was demonstrated.

## 1. Introduction

The main problem of recent times is environmental pollution. This is related to, among others, an increase in the production and consumption of polymer materials by an average of 9% per year and an assumption that the upward trends will be continued (it is estimated that, in the coming years, the increase will be 5% a year [1]). According to Plastics Europe [2], world polymer production increased from 1.5 million tonnes in 1950 to 245 million tonnes in 2008. The European Union (EU) economy produces around 20% of the total world polymer production. The demand for raw materials in individual European countries strongly depends on the size of the given economy and the degree of its development. For example, economically leading countries such as Germany, Italy, France, and the United Kingdom consume as many polymer materials as all other European countries put together [3]. According to Plastic Europe Polska [4], however, consumption has been constant for individual countries in recent years. Only the demand for individual types of polymer materials is subject to fluctuations [5]. An exception is, among others, Poland, where demand increases every year, regardless of the global economic situation [4]. According to Forbes [6], the global demand for certain plastic uses has increased owing to the threat posed by coronavirus. These include mainly polymer polypropylene, used in takeout food packaging, and polyethylene terephthalate (PET) in single-use plastic water bottles. This was the result of the shift from sit-in restaurants to take-out delivery and of the stockpiling of groceries and bottled water by consumers. For the same reason, the amount of glass waste increased, which is one of the most common materials in everyday life. The recycling rate of glass waste is quite low in many countries, compared with other solid wastes [7]. For example, in the United States, 11.38 million tonnes of waste glass were produced in 2017, but 26.6% was recycled and mainly used for the production of containers and packing, and 60.37% was landfilled [8]. In Hong Kong, 4063 and 7174 tonnes of glass waste was generated in 2018 and 2019 respectively, and the recovery rate was about 16.3% in 2018. The total amount of used glass containers that ended up in landfills in 2018 was 77,400 tonnes [9]. In Singapore, 72.8 million tonnes of glass were disposed in 2011, but only 29% was recycled [7]. In the United Kingdom, 1.85 million tonnes of waste glass are collected annually, and for container glass, the municipal recycling rate is 34% [10].

Concrete is a material that allows for the disposal of waste [11,12,13,14,15,16,17,18,19,20]. This is very important in the case of non-biodegradable or hardly decomposable materials such as polymers or glass waste. Polymer materials have been used as a replacement for natural aggregate in concrete [21,22,23,24,25], replacement of cement [26,27,28], additions (e.g., PET bottles [29,30,31], polyvinyl chloride (PVC) pipes [32], high density polyethylene (HDPE) [33], and thermosetting plastics [34]), expanded polystyrene foam (EPS) [35], polypropylene fibres [31,36,37], admixtures (e.g., polycarbonate and polyurethane foam [38,39,40]), or elements to concrete (e.g., concrete reinforcing bars [41] and plastic anchors [3,42,43]). The used polypropylene fibre by-products of recycling plastic packaging in concrete compared with plain concrete have been discussed in detail in a previous study [44]. Glass cullet may be used as a replacement for cement or aggregate [45,46,47,48], while the pozzolanic reactivity of glass powder with particle size below 100 μm is observed as an increase in compressive strength [49,50,51]. The impact of glass powder as a cement replacement on concrete or geopolymer properties was presented in [52,53,54,55,56]. For example, Federico and Chidiac [57] analysed the kinetic and performance properties of cementitious mixes with glass powder. Mirzahosseini and Riding [58] investigated the impact of curing temperature and glass type on the pozzolanic reaction and properties of concrete with glass powder.

Many scientists have tested concrete with glass aggregate as a replacement of coarse aggregate, fine aggregate, or cement in order to use waste glass in the concrete industry [59,60,61]. Yu et al. [62] reported that the glass cullet used as aggregate in concrete enhanced its mechanical properties. Limbachiya et al. [63] and Tittarelli et al. [64], however, obtained the same mechanical performances for concrete mixes with addition of glass sand up to 15%. It was found that the use of glass cullet as replacement for coarse aggregate is not satisfactory owing to the reduction of the bonding between the aggregate and the cement matrix, and a reduction of strength [65]. The effect of the size of glass particles on the properties of fresh mix and hardened samples was analysed by Ling and Poon [66] and Yousefi et al. [67]. However, the impact of fibres on the properties of a cement–glass composite has rarely been reported.

On the other hand, the production of building materials is responsible for significant energy consumption and CO_2_ production [68,69,70], so it is necessary to look for new materials that can replace the currently used ones [71,72], preferably recycled [73,74]. The addition of recycled glass aggregate in concrete as a replacement of 5%, 10%, and 15% natural aggregate has been studied in previous research of the authors [75]. In this study, 100% of glass aggregate is used, which is a continuation and extension of research conducted by Małek at el. [74]. Moreover, as the cement–glass composite has low tensile strength and brittle failure, polypropylene fibres are additionally added to improve its tensile strength. In order to contribute to the use of recycled materials, polypropylene fibres were made from post-consumer waste (food packaging). This research aims to assess the influence of different fibre content on the mechanical properties of the cement–glass composite.

## 2. Materials

### 2.1. Products of Cementitious Mix

**Figure 1:**
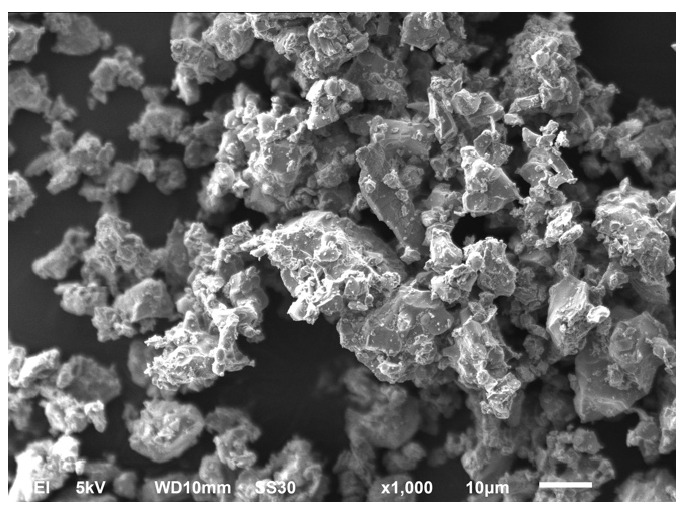
Scanning electron microscopy (SEM) image of used cement.

In this research, Portland cement, tap water, and polycarboxylate superplasticizer were used. The concept of designing a cement–glass composite is based on a single binder in the form of white cement. Because of the fact that the composition of the composite consisted of a 100% granulated glass cullet, the industrial white Portland cement CEM I 52.5R NA, pH = 13 was used. It is a special purpose cement with a very low content of alkaline compounds. The chemical composition of cement was investigated by PN EN 196-6:2011 [76] and is presented in Table 1. The physical properties and compressive strength of cement were determined according to PN-EN 196-6:2011 [76] and PN EN 196-1:2016-07 [77], respectively (see Table 2). The shape and texture of cement gain particle were investigated by scanning electron microscopy (SEM—Joel JSM 6600, Yvelines, France), as shown in Figure 1.

**Figure 2:**
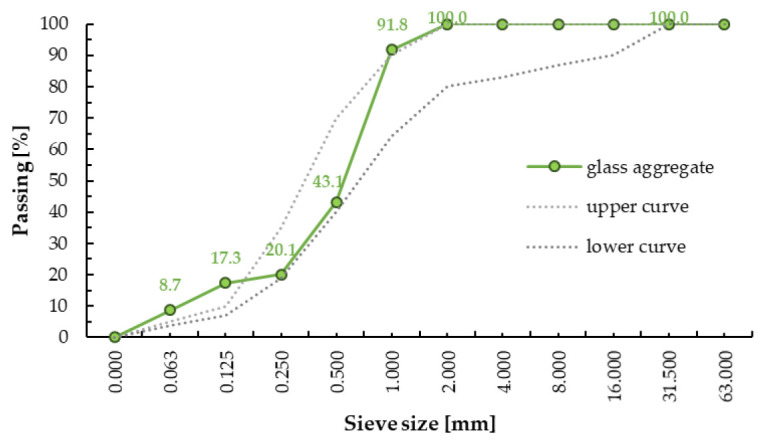
Gradation curve of glass aggregate.

**Figure 3:**
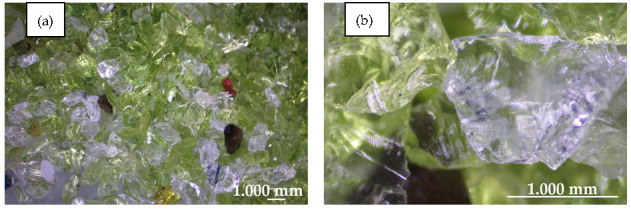
Light microscope images of used glass aggregate. (**a**) magnification of x5; (**b**) magnification of x45.

Recycled sodium glass granules were used as aggregate in the composition of the composite. Sieve analysis was performed using the “dry” method for three samples of the granulate using a laboratory shaker with a set of standard sieves with square meshes made of calibrated mesh. The percentage distribution of individual fractions of screened granules was determined. The crumb pile was designed from two granules of the fraction groups 0/0.9 (0/1) and 0.9/1.5 (1/2). As an additive in the composition of the composite (filler), glass powder from sodium glass with a particle size from 0 to100 µm and dry density of 1.0 kg/m^3^ was used. The glass powder acts as a sealer for the crumb pile, the purpose of which is to ensure the continuity of the internal structure of the material. The graining curve was designed following the graining guidelines for sand concrete; the designed curve was related to the upper and lower limit curves. The designed curve ran in the area of good particle size distribution. The gradation curve of the used glass aggregate is presented in Figure 2. The crushed glass cullet revealed sharp edges, a rougher surface texture, and no cracks (Figure 3). The specific density and Mohs hardness scale of the glass aggregate was approximately 1.6 kg/m^3^ and 6–7, respectively. The fineness modulus of the glass sand aggregate was equal to 2.56 MPa. The chemical composition and physical properties of glass cullet are given in Table 1 and Table 2, respectively.

**Figure 4:**
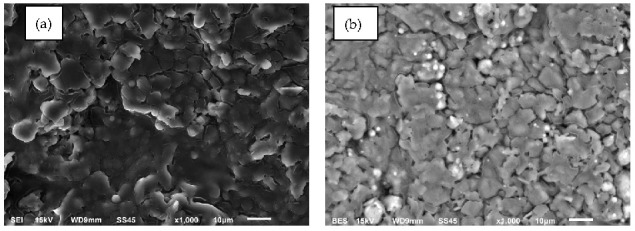
SEM microstructures of admixture. (**a**) SE mode; (**b**) BSE mode.

A superplasticizer was used in this research based on modified polycarboxylate ethers (melamine and silanes/siloxanes). The superplasticizer was added to reduce the amount of water (maintaining a water/cement ratio w/c at 0.26). The particle shape and texture of the admixture were investigated by scanning electron microscopy (Joel JSM 6600, Yvelines, France), as shown in Figure 4.

### 2.2. Polypropylene Fibres

**Figure 5:**
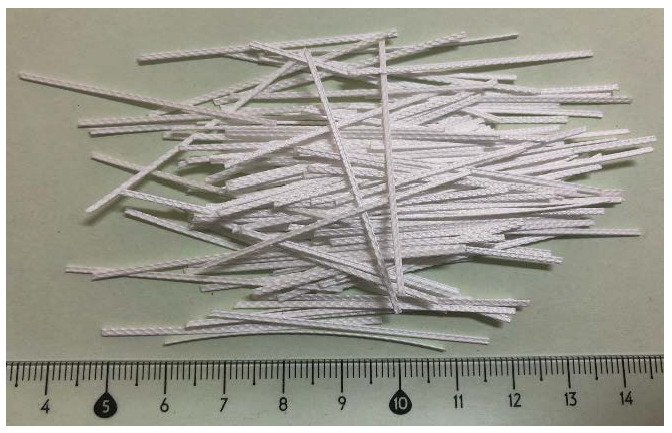
Polypropylene recycled fibres (PPW) with length ranging from 27.1 to 32.6 mm.

**Figure 6:**
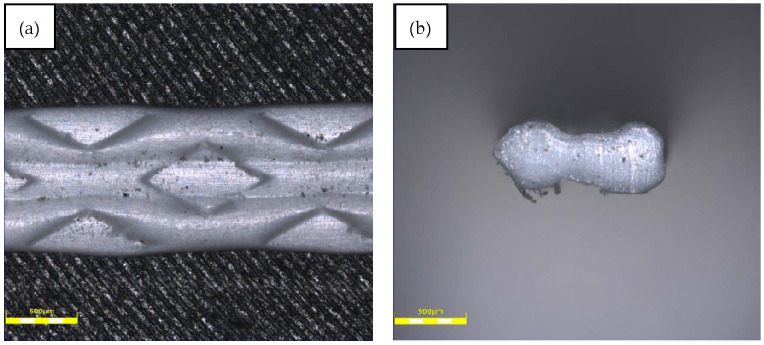
Light microscope images of the used fibres: (**a**) surface and (**b**) cross section.

**Figure 7:**
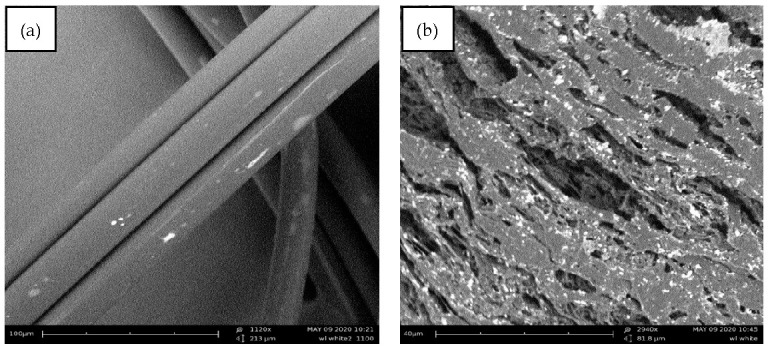
SEM images of the used fibres: (**a**) surface and (**b**) cross section.

Polypropylene fibres made from waste materials (plastic packaging) were used (Figure 5). The white polypropylene fibres (PPW) were made through a cutting process and their surface was modified into the extruder to increase its adhesion to the cementitious mix. Because of this modification process, the surface of PPW is irregular (Figure 6 and Figure 7).

Polypropylene fibres of 31.2 ± 0.5 mm in length and 1152.5 ± 10.0 μm in diameter were used. The average circumference was 538.2 ± 0.5 μm. The tensile strength of the fibres was about 520 MPa, the modulus of elasticity was about 7.5 GPa, and Poisson ratio was about 0.2. Fibre content ratios of 300, 600, 900, 1200, and 1500 g/m^3^ were used.

### 2.3. Mix Composition

Six different modifications of concrete mixtures (reference—without fibres, and five with fibres) were produced. The polypropylene fibre content was about 0.0625%, 0.1250%, 0.1875%, 0.2500%, and 0.3125% of cement mass, respectively; see Table 3. A constant water to cement ratio, *w_eff_/c* = 0.29, was used for all mixes, where *w_eff_* was tap water content and *c* was the cement content. The admixture amount was 1.0% of the cement mass, which corresponds with [75].

### 2.4. Mix Production

The technology of mixing individual components of the composite assumes using the “dry” and “wet” mixing methods. For the mixing stage, a high-speed planetary mixer (mixer) with variable speeds of stirrer rotation (three ranges of stirrer rotation speed) was used. Various mixing speeds and types of agitator were used. Two types of mixers were used: flat and “hook”. The appropriate types of mixers were selected depending on the sequence of ingredients and their type. The following sequence and methods of mixing the composite components were proposed:

- Glass granulate of 0.9/1.5 mm fraction and a group of granulate fraction 0/0.9 mm—“dry” mixing, mixing time 2 min from the moment of filling the mixing container with the above-mentioned granulate fractions, mixing speed: gear 1, type of agitator: flat;
- Cement binder with filler (glass powder 0/200 µm)—“dry” mixing, mixing time: 2 min from the moment the mixing container is filled with the listed ingredients, mixing speed: gear 1, type of agitator: flat;
- Mixing water with liquid chemical admixture—mixing time: 2 min from the moment of adding both components to the measuring cylinder, mixing speed: gear 1 (mixing takes place with the measuring cylinder by means of a stirrer), type of stirrer: magnetic;
- Glass granulate with cement and glass powder—mixing time: 3 min from the moment of adding all ingredients to the mixing container of the mixer, mixing speed: gear 1, type of agitator: “hook”, dry mixing;
- Glass granulate with cement and glass powder—mixing time: 3 min, mixing speed: gear 2, type of agitator: flat, mixing speed: gear 1, “dry” mixing;
- Glass granulate with cement and glass powder and mixing water with a liquid chemical admixture—mixing time: 3–4 min, mixing speed: gear 1, type of agitator: flat, “wet” mixing;
- Glass gargoyle with cement and glass powder, and mixing water with a liquid chemical admixture—mixing time: 3 min, mixing speed: gear 2, type of agitator: flat, “wet” mixing;
- Glass granulate with cement and glass powder and mixing water with a liquid chemical admixture—mixing time: 3 min, mixing speed: gear 3, type of agitator: flat, “wet” mixing.

## 3. Methodology

### 3.1. Test on Mix

Slump cone and air content were measured after production of mixture. The slump cone was investigated according to PNEN 12350-2:2019-07 standard [80]. In order to preserve the statistics of the results, measurements were made on five test samples for each mixture. In order to obtain the air content, the pressure method was investigated per ASTM C231 standard [81]. Five samples were used for each mixture.

### 3.2. Test on Hardened Concrete

After 28 days of curing, the hardened concrete was investigated. For this purpose, five samples were measured for each concrete mixture. In order to determine the mechanical properties of the manufactured concrete, ten specimens were used for each test.

#### 3.2.1. Material Properties

The density of the prepared concrete samples (150 mm × 150 mm × 150 mm) was measured according to the standard PN-EN 12390-7:2011 [82].

#### 3.2.2. Mechanical Properties

The mechanical properties of hardened concrete were measured using three methods: compressive strength, splitting strength, and flexural strength. For each test, the Zwick machine was used with a force range of 0–5000 kN (Zwick, Ulm, Germany). In addition, the modulus of elasticity and Poisson ratio were investigated.

1. *Static compression test of cubic samples*

The compressive strength test was carried out on cubic samples with dimensions of 150 mm × 150 mm × 150 mm according to the standard EN 12390-3:2019-07 [83] after 28 days of curing under standard conditions (21 °C, 50% humidity). Cubic samples, after being removed from the care bath, were dried of excess water. Compressive strength tests were carried out 30 min after the end of curing. The samples were placed in the vertical working space on the lower clamping plate.

1. *Static flexural test of beam samples*

A static bending test of beam samples was carried out in order to determine the flexural strength of the composite modified with waste fibres. Specimens with dimensions of 100 mm × 100 mm × 500 mm were prepared and subjected to a three-point flexural test on a testing machine according to the standard EN 12390-5:2019-08 [84]. The beams were fixed on supports with movable rollers and then loaded with concentrated force on the middle of the span. The spacing in the axes of the supports was set at 400 mm. The construction of supports and concentrated forces with movable rollers eliminated the negative effect of frictional forces on the course of testing beam samples. On the basis of the static three-point flexural test, the values of the maximum destructive force and the maximum destructive stresses were recorded.

1. *Static splitting test of cubic samples*

The splitting strength of the composite was determined by the splitting method according to EN 12390-6:2011 [85], the so-called “Brazilian” method. The test was carried out after 28 days of maintenance. The surfaces of the samples were cleaned of the sediment after the treatment was completed and the excess surface water was dried. The cubic composite samples were placed in a metal splitting test profile. The rate of stress increase in time was determined as 0.5 MPa/s. A static sample splitting test was performed. Based on the registered maximum failure force in the compression test, the specified value of failure stress was determined.

1. *Modulus of elasticity and Poisson coefficient*

The study of Young’s modulus was performed with a non-destructive method per ASTM C215-19 standard [86] using the James TM E-Meter Mk II apparatus (Chicago, IL, USA), which uses principles based on determining the basic resonance frequencies of vibrations generated by the shocks measured by the accelerometer. This device tests three types of vibrations: longitudinal, transverse, and torsional. The method was applied on cylindrical samples with a diameter of 150 mm and a height of 300 mm after at least 28 days of hardening.

## 4. Results and Discussion

### 4.1. Fresh Properties

Table 4 presents the results of fresh mix properties. The average values of the five samples for each mix are given.

**Figure 8:**
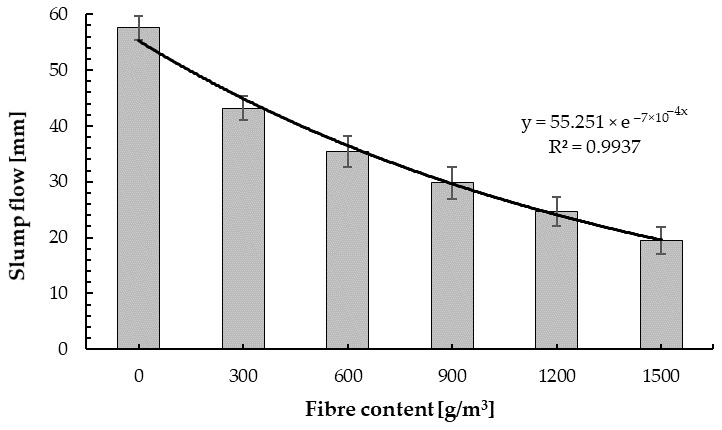
Slump test results.

During the mixing, no process of agglomeration formation of fibres was observed for all cement–glass composite mixes, which is a main problem of concrete mixes with fibres. The fibres did not float to the surface nor did they sink to the bottom in the fresh mixes. They were mixed smoothly with the composite mixture. Figure 8 presents the results of the slump cone (SC) test. It can be observed that, by replacing the entire aggregate with glass aggregate and using glass powder as a filler [87], the reference mix and the mix with a lower fibre content were within slump class S2 [80]. Moreover, the slump cone obtained for the cement–glass composite was about 66% higher than for plain concrete with granite aggregate and a strength of 50 MPa [74]. An increase in slump with increasing fine glass aggregate was observed by Castro and Brito [88], while according to Limbachiya [89] and Taha [90], the use of glass sand resulted in the decrease in the workability of the concrete due to a lack of fine proportion.

The addition of PP fibres to the reference mix reduces its workability, which is analogous to observations for plain concrete reinforced PP fibres [44]. The mixes with 600 to 1500 g/m^3^ of fibres were within slump class S1 [80]. Moreover, with the addition of 300, 600, 900, 1200, and 1500 g/m^3^ of fibres, the decrease in slump was 25.9%, 39.7%, 48.3%, 56.9%, and 65.5%, respectively, compared with the reference specimen (without fibres). Practically, the same decrease in slump cone was observed for plain concrete reinforced with polypropylene fibres made from plastic packaging with the same contents [44]. A similar correlation of reduction in workability with an increase in fibre content was determined by other scientists [91,92].

The air content of the cement–glass composite was constant regardless of the PP fibre content and was equal to 4.0 ± 0.5% (Table 4). The addition of fibres did not affect the air content in the mixture. The obtained air content of the cement–glass composite, however, was two times higher than for plain concrete and for concrete with the addition of glass aggregate up to 20% of cement [74].

### 4.2. Hardened Properties

#### 4.2.1. Density

**Figure 9:**
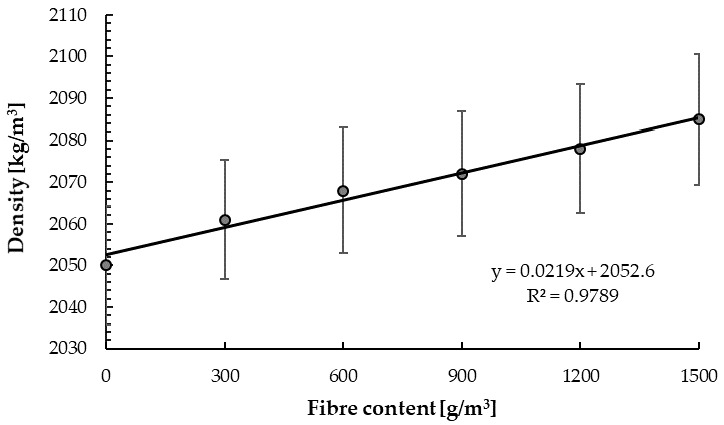
The correlation between the amount of fibres and the density of the cement–glass composite.

The results of cement–glass composite density are given in Figure 9 and Table 5. The presented values are the average values of the five samples for each mix for density and ten samples for mechanical properties.

**Figure 10:**
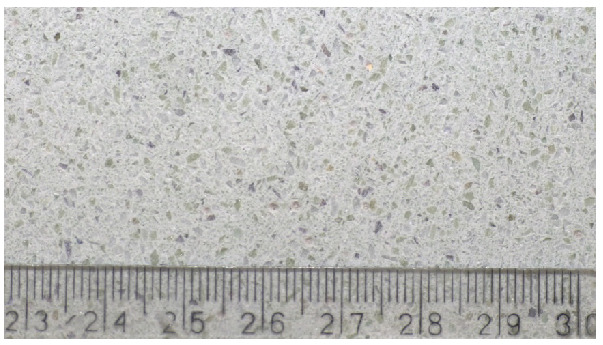
Distribution of components in the cement–glass composite.

With the increase in fibre content, the cement–glass composite density increased linearly (Figure 9), while the impact of glass sand addition was negligible. For the highest fibre content, the composite density increased by 1.7% compared with the base sample. This could be related to the composite production error or the occurring pozzolanic reaction [93]. Figure 10 presents the uniform distribution of glass aggregate and fibres in the sample.

The obtained cement–glass composite density was much lower than the density of plain concrete with granite aggregate (*γ* = 2205 ± 4 kg/m^3^) and follows the demonstrated trend of the decrease in density with increasing glass fine aggregate [74]. Other scientists [94,95] observed the same results. Park et al. [95] obtained a linear decrease of concrete density with increasing waste glass aggregate content, while Lee et al. [94] determined that replacing the aggregate with glass sand up to the amount of 20–25% slightly affects concrete density.

#### 4.2.2. Compressive Strength

**Figure 11:**
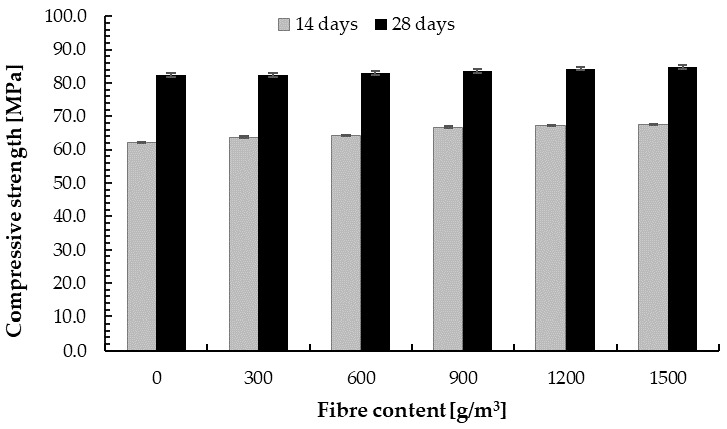
Compressive strength of the cement–glass composite depending on fibre content.

The test results of compressive strength for samples of the cement–glass composite are shown in Figure 11. The compressive strength increased slightly with the increase in PP fibre addition, which was unexpected owing to the principle that fibres improve tensile strength, but not compressive strength [96,97,98]. Oni et al. [99] determined a slight increase for concrete with 0.3% PP fibre and a slight decrease with the addition of 0.4% of this fibre. For other tested types of polypropylene fibre, they obtained decrease in compressive strength. Moreover, Jiang et al. [100] reported 3.12% decrease in the compressive strength of PP fibre-reinforced concrete compared with the base sample.

It can be observed that, after 14 days, 75% of the compressive strength after 28 days was obtained. Moreover, the compressive strength of the cement–glass composite was 1.5 times higher than for plain concrete of similar composition (cement CEM I 52.5R, granite aggregate 0/4 mm, and with the addition of a superplasticizer to reduce the amount of water to w/c = 0.26), for which the compressive strength was *f_c_* = 53 MPa [74]. This is probably the result of the use of glass powder to ensure the continuity of the internal structure of the material, which in turn results in an increase in mechanical strength by reducing the air pores in the cement hydration process. In addition, according to observations of other scientists, the pozzolanic reactivity of fine waste glass with a particle size below 100 μm is observed as an increase in compressive strength [49,50,51] due to pozzolanic reaction. Moreover, this is in line with the observation that compressive strength improves with increase in the content of glass fine aggregate [74,101,102]. Lee et al. [94] obtained a 34.3% increase in compressive strength compared with plain concrete for concrete with fine glass aggregate with a particle size of 0–0.6 mm. Chung et al. [103] also reported that the use of aggregates with size less than 4.0 mm makes it possible to achieve an improvement in compressive strength. Using glass powder in concrete mixture could result in higher compressive strength, as increases in the compressive strength of concrete with glass powder have been found in other studies. Bajad et al. [104] determined that compressive strength increases with the addition of glass powder as a cement replacement at a ratio of up to 20%, and then decreases. Improvements in the long-term compressive strength of concrete containing fine glass powder have been reported by other scientists [36,105]. The increase in compressive strength could be caused by the pozzolanic reaction of very fine particles [57,90,106,107]. According to Shi et al. [108] the strength activity indices of fine glass powder with a size of 40–700 μm were 70% to 74% at 7 and 28 days, respectively. Kamali and Ghahremaninezhad [109] and Ling and Poon [66] reported that smaller particle sizes of aggregate enhance the aggregate–cement matrix bonding strength. Yamada et al. [110] demonstrated the critical particle size to range from 0.15 to 0.30 mm for the occurring pozzolanic reaction, while Jin et al. [111], Idir et al. [112], and Xie et al. [113] determined this size to be from 0.60 to 1.18 mm.

#### 4.2.3. Flexural Strength

**Figure 12:**
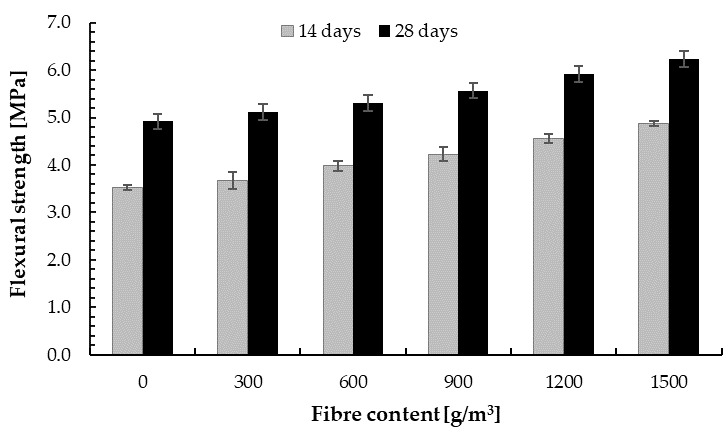
Flexural strength of the cement–glass composite depending on fibre content.

The results of the flexural test of the cement–glass composite are shown in Table 5 and Figure 12. It can be observed that, with the addition of 300, 600, 900, 1200, and 1500 g/m^3^ of PP fibre, flexural strength increased compared with the base sample by 4.1%, 8.2%, 14.3%, 20.4%, and 26.5%, respectively. These values were obtained for a very small amount of fibre ranging from 0.0625% to 0.3125% of the cement weight. Thus, the addition of polypropylene fibre improves the flexural strength of the cement–glass composite. This is analogous to fibre-reinforced concrete with natural aggregate [95,114,115]. Nili and Afroughdaset [116] obtained a 22% improvement for concrete with silica fume and 0.3% of PP fibre. Satisha et al. [117] also determined about a 30% increase in flexural strength for concrete with 2.0% addition of PP fibres. About a 37% increase in flexural strength for concrete with 1.0 wt.% polypropylene fibres was obtained by Badogiannis et al. [118]. Other scientists reported an increase in flexural strength with addition of PP fibre ranging from about 10% [119,120,121] to 35% [118]. A higher increase in flexural strength was observed in the literature, but for significant proportions of PP fibres [121,122,123].

Moreover, after 14 days of curing, flexural strength ranging from about 70% to 80% of the target flexural strength was obtained. The increase in 14-day flexural strength was 5.7%, 14.3%, 20.0%, 31.4%, and 40.0%, respectively, compared with the base sample. This proves the large use of recycled fibres and is a continuation of the research presented by Malek et al. [44].

The flexural strength of the cement–glass composite was almost half that of the flexural strength of plain concrete of a similar composition, but with granite aggregate (*f_tk_* = 10.5 ± 0.3 MPa [74]). According to Tan and Du [112], the reduction in flexural strength is caused by a decrease in adhesive strength at the glass particle surface and cement matrix and, additionally, micro-cracks in the case of clear glass aggregate. The effect of the weaker bonding of glass aggregate with the cement matrix is more important in the flexural test than in the compression test. Moreover, this runs contrary to the observation that bending strength is enhanced with the addition of fine glass aggregate, and thus the increase in fracture toughness [24,74]. The reduction in flexural strength, however, was demonstrated by other scientists [103]. Ling and Poon [66] obtained about a 30% decrease in flexural strength for concrete with 100% glass aggregate (60 wt.% glass aggregate size from 0 to 2.36 mm and 40 wt.% size from 2.36 to 5.00 mm).

#### 4.2.4. Splitting Strength

**Figure 13:**
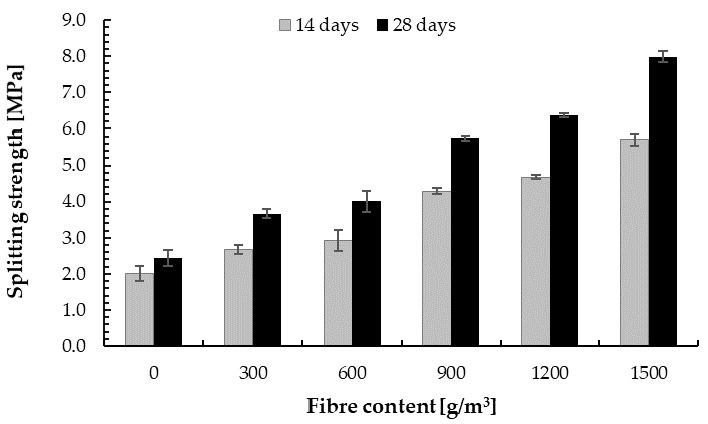
Splitting strength of the cement–glass composite depending on fibre content.

Table 5 presents the splitting strength for the cement–glass composite with different fibre content. With the increase in PP fibre content, the splitting strength increased linearly; see Figure 13. For the cement–glass composition with 300, 600, 900, 1200, and 1500 g/m^3^ of PP fibre, the 28-day splitting strength was 35%, 45%, 115%, 135%, and 185% higher, respectively, than for the reference sample, while the increase in the 14-day splitting strength was 48%, 68%, 132%, 156%, and 220%, respectively, compared with the base sample. This significant improvement was obtained for a very small amount of fibre ranging from 0.0625% to 0.3125% of the cement weight. Thus, the addition of polypropylene fibre improves the splitting strength of the cement–glass composite. The same phenomenon was observed by other scientists for concrete with natural aggregate [95,124,125]. The fibres are able to bridge the cracks and transfer stress across the cracks [126,127]. The fibre-reinforced composite is destroyed when the fibre slides out of the matrix or breaks (in the second case, the load is redistributed to the other fibers [128]). Thus, the method of damage of the fibre-reinforced cement–glass composite mostly depends on the strength of the materials and the adhesion of the fibers to the matrix [129,130,131].

The increase in splitting strength was much larger than the increase in flexural strength. Analogous to flexural strength, however, after 14 days of curing, splitting strength from about 70% to 80% of the target flexural strength was obtained.

The splitting strength of the cement–glass composite was about two times lower than for plain concrete of a similar composition, but with granite aggregate (*f_r_* = 4.12 MPa [74]). With the addition of glass aggregate, the splitting strength improved compared with the base sample [7,74]. The sample with 100 wt.% of glass sand aggregate, however, was lower. This may indicate that the strength increases with increasing cullet content and then decreases.

#### 4.2.5. Modulus of Elasticity and Poisson Ratio

The results of the modulus of elasticity are shown in Table 5. In this study, the modulus of elasticity obtained was about 31 ± 1 GPa regardless of fibre content, and was equal to normal concrete with granite aggregate (*E* = 32 ± 1 GPa [74]). According to other papers [74,98], an insignificant effect of glass aggregate content on the elastic modulus can be observed.

The addition of PP fibre up to 0.3125% of the cement did not affect the Poisson ratio (Table 5).

## 5. Conclusions

The purpose of the research was to assess the possibility of using a large amount of glass cullet as a substitute for concrete components. Glass powder as filler and 100% of glass aggregate were used. The cement–glass composite exhibited low tensile strength and brittle failure. In order to improve tensile strength, the effects of adding polypropylene fibres on the mechanical properties of the composite were examined. The polypropylene fibre content was 0.0625%, 0.1250%, 0.1875%, 0.2500%, and 0.3125% of cement mass, respectively. Based on the results of this experimental investigation, the following key conclusions can be drawn:

- An effect of a decrease in the slump cone with the addition of PP fibres was noted; the reference mix and the mix with a lower fibre content were within slump class S2, but the mix with higher PP fibre content was within slump class S1.
- The amount of air in the cement–glass composite mix was equal to 4.0 ± 0.5%. The addition of fibres did not affect the air content of the mixture.
- With the increase of PP fibre content, the density of the cement–glass composite increased, but this effect was negligible (2–3% compared with the reference sample).
- With the addition of 0.0625%, 0.1250%, 0.1875%, 0.2500%, and 0.3125 wt.% polypropylene fibre, the increase in flexural strength of the cement–glass composite compared with the reference sample was about 4%, 8%, 14%, 20%, and 27%, respectively, while the increase in splitting strength was about 48%, 60%, 132%, 156%, and 220%, respectively. The effect of the increase in splitting strength was much larger than the increase in flexural strength. The compressive strength increased slightly with the increasing PP fibre content, which was unexpected owing to the principle that fibres improve tensile strength, but not compressive strength (0.1%, 0.6%, 1.6%, 2.3%, and 2.8% increase for 0.025, 0.050, 0.075, 0.100, and 0.125 wt.% polypropylene fibre, respectively).
- The elastic modulus of the cement–glass composite with content of 0.0625%, 0.1250%, 0.1875%, 0.2500%, and 0.3125 wt.% PP fibre was about 31 ± 1 GPa regardless of fibre content, and was equal to plain concrete with granite aggregate.
- The addition of PP fibre up to 0.3125% of the cement did not affect the Poisson ratio.

High values of flexural and splitting strength are the results of polypropylene fibres. This research will be subject to further testing. Other types of cement, glass waste and its mixes, and different contents of glass powder, with particular emphasis on long-term fatigue tests, are planned in this respect. In addition, it should be emphasized that the modulus of elasticity of the tested concrete composite is very low, which may result in greater deflection of the structure. This is why elements with no significant deflections, such as columns or sheet piling, can be made of a cement–glass composite (preferably with the highest obtained mechanical properties, e.g., recipe M5 with addition of 0.3125% PP fibres).

## Figures and Tables

**Table 1:** Chemical composition of cement and glass cullet [78,79].

Compositions		SiO_2_	Al_2_O_3_	Fe_2_O_3_	CaO	MgO	SO_3_	Na_2_O	K_2_O	TiO_2_	Cl
Unit (vol.%)	Cement	19.5	4.9	2.9	63.3	1.3	2.8	0.1	0.9	-	0.05
	Glass	70.0–74.0	0.5–2.0	0.0–0.1	7.0–11.0	3.0–5.0	-	13.0–15.0		0.0–0.1	-

**Table 2:** Physical properties of cement and glass cullet [79].

Properties	Specific Surface Area [m^2^/kg]	Specific Gravity [kg/m^3^]	Compressive Strength after Days [MPa]		
Materials			2 Days	7 Days	28 Days
Cement	400	3090–3190	40–48	53–65	66–76
Glass	100	2450	-	-	-

**Table 3:** Mix proportions (1 m^3^).

Mix Symbol	Cement [kg]	Water [kg]	Glass Powder [kg]	Glass Sand Aggregate		Fibre [g]
				0.1–0.9 mm	0.9–2.0 mm	
M0	480	140	600	510	790	0.0
M1						300
M2						600
M3						900
M4						1200
M5						1500

**Table 4:** Fresh properties.

Mix Symbol	Fibre Content [g/m^3^]	Slump Cone [mm]	Air Content [%]
M0	0	58 ± 2	4.0 ± 0.5
M1	300	43 ± 2	3.8 ± 0.3
M2	600	35 ± 3	3.9 ± 0.2
M3	900	30 ± 3	3.5 ± 0.5
M4	1200	25 ± 3	3.6 ± 0.6
M5	1500	20 ± 3	3.6 ± 0.6

**Table 5:** The results of the properties of hardened samples.

Mix Symbol	Density [kg/m^3^]	Compressive Strength [MPa]		Flexural Strength [MPa]		Splitting Strength [MPa]		Elastic Modulus [GPa]	Poisson Ratio [[–]
		14 Day	28 Day	14 Day	28 Day	14 Day	28 Day		
M0	2050 ± 14	62.1 ± 0.8	82.5 ± 0.8	3.5 ± 0.1	4.9 ± 0.2	2.0 ± 0.7	2.5 ± 0.8	31 ± 1	0.15 ± 0.01
M1	2061 ± 14	63.8 ± 0.9	82.6 ± 0.5	3.7 ± 0.6	5.1 ± 0.1	2.7 ± 0.4	3.7 ± 0.4	32 ± 1	0.15 ± 0.02
M2	2068 ± 15	64.2 ± 0.3	83.0 ± 0.8	4.0 ± 0.3	5.3 ± 0.1	2.9 ± 0.9	4.0 ± 0.8	32 ± 1	0.15 ± 0.02
M3	2072 ± 15	66.8 ± 0.8	83.8 ± 0.3	4.2 ± 0.5	5.6 ± 0.1	4.3 ± 0.2	5.8 ± 0.3	32 ± 1	0.15 ± 0.01
M4	2078 ± 15	67.3 ± 0.3	84.4 ± 0.6	4.6 ± 0.3	5.9 ± 0.1	4.7 ± 0.1	6.4 ± 0.6	32 ± 2	0.15 ± 0.02
M5	2085 ± 16	67.6 ± 0.6	84.8 ± 0.6	4.9 ± 0.1	6.2 ± 0.2	5.7 ± 0.5	8.0 ± 0.6	32 ± 1	0.15 ± 0.01

## Data Availability

Data is contained within the article.
